# Critical examination of knowledge to action models and implications for promoting health equity

**DOI:** 10.1186/s12939-015-0178-7

**Published:** 2015-05-29

**Authors:** Colleen M. Davison, Sume Ndumbe-Eyoh, Connie Clement

**Affiliations:** Department Public Health Sciences, Queen’s University, 63 Fifth Field Company Lane, Kingston, ON K7L 3N6 Canada; National Collaborating Centre for Determinants of Health, St Francis Xavier University, P.O. Box 5000, Antigonish, NS B2G 2WG Canada

**Keywords:** Health equity, Knowledge translation, Public health interventions, Health inequalities

## Abstract

**Introduction:**

Knowledge and effective interventions exist to address many current global health inequities. However, there is limited awareness, uptake, and use of knowledge to inform action to improve the health of disadvantaged populations. The gap between knowledge and action to improve health equity is of concern to health researchers and practitioners. This study identifies and critically examines the usefulness of existing knowledge to action models or frameworks for promoting health equity.

**Methods:**

We conducted a scoping review of existing literature to identify knowledge to action (KTA) models or frameworks and critiqued the models using a health equity support rubric.

**Results:**

We identified forty-eight knowledge to action models or frameworks. Six models scored between eight and ten of a maximum 12 points on the health equity support rubric. These high scoring models or frameworks all mentioned equity-related concepts. Attention to multisectoral approaches was the factor most often lacking in the low scoring models. The concepts of knowledge brokering, integrative processes, such as those in some indigenous health research, and Ecohealth applied to KTA all emerged as promising areas.

**Conclusions:**

Existing knowledge to action models or frameworks can help guide knowledge translation to support action on the social determinants of health and health equity. There is a need to further test existing models or frameworks. This process should be informed by participatory and integrative research. There is room to develop more robust equity supporting models.

**Electronic supplementary material:**

The online version of this article (doi:10.1186/s12939-015-0178-7) contains supplementary material, which is available to authorized users.

## Introduction

Each year, preventable disease and disability and shortfalls in the determinants of health take the lives and diminish the health and well-being of millions of people globally [1, 2]. Inequalities in health and social circumstances across populations persist within and between countries and regions. Groups experiencing social and economic exclusion that leads to unequal access to health and its determinants include, for instance, people living in poverty, people with disabilities, racialized peoples, and Indigenous peoples who are disproportionately affected by poor health and shorter lives [3]. The resulting health inequities are differences in health that are judged to be unfair or the result of some form of historical or contemporary injustice [4]. They have also been defined as systematic, unfair and avoidable inequalities [5]. Striving for health equity means working so that everyone can reach their full health potential and not be disadvantaged from attaining this because of their class, socioeconomic status or other socially determined circumstance [6, 7].

Different forms of knowledge and effective interventions exist to address many health concerns and inequalities however, awareness, uptake and use of these can be poor and poorer still with respect to interventions to improve health across social gradients [8].

The difference between what is known about a particular health issue or possible intervention, and what is being done for health promotion and disease prevention is termed the “know-do gap”. This gap between knowledge and action is of prime concern to public health researchers and practitioners, and pertains increasingly more prominently to what is not being done to improve health equity including improving health for disadvantaged populations. “Knowledge to action” is a broad term used to refer to the process of bridging the know-do gap. This concept has been described in many different ways including the translation, dissemination, implementation, transfer and exchange of knowledge, the diffusion of an innovation or idea, and the use of knowledge or research evidence to inform decision making [9–11]. Knowledge to action scholarship has increased significantly over the past 20 years [6, 12, 13]. In 1990, fewer than a hundred articles were retrieved in a knowledge translation keyword search in Medline. In February 2006, several thousand articles were found with the same search strategy [14]. In August 2012, we retrieved nearly 110,000 articles with the keywords: knowledge translation, knowledge transfer, dissemination, evidence-based, and knowledge to action. While there have been many papers on knowledge to action, there has been limited research that explores which strategies or components may be most effective for supporting health equity [15]. The evaluations that do exist have primarily occurred for evidence-based medical practice in high-income countries [16]. There is significant potential for knowledge to action theory, models, frameworks and methodologies to contribute to the discourse, and inform action to address health inequities more widely [17].

In the past few years, there have been key publications such as *Closing the Gap in a Generation: Health equity through action on the social determinants of health* [1] and *Integrating Social Determinants of Health and Health Equity Into Canadian Public Health Practice* [2] that have specifically called for bridging the know-do gap for health equity gains. Knowledge to action approaches can bridge the gap between what we know about health inequities and what is being done to address and reduce them. Specifically, there is a need for an explicit focus on equity in decision and policy making, for instance with respect to distribution of resources, prioritization of issue and targeting of interventions Inclusive and participatory approaches recognizing varied forms of knowledge and perspectives; interaction across jurisdictions and sectors; and consideration of the social, political and economic factors that support or deter efforts towards health equity are required at many levels of decision making [1, 2].

The purpose of this analysis was to identify existing knowledge to action models or frameworks and critically examine a promising subset of them as to their utility for promoting or supporting health equity.

## Methods

A scoping review was conducted by CMD and a research associate Ariel Pulver (AP) for preexisting knowledge to action models or frameworks. This was not designed to be an exhaustive review, but to generate a group of recent models or frameworks that could be assessed in relation to health equity. Given the numerous conceptualizations and diverse terminologies [9] used in the field, the search and subsequent accumulation of models or frameworks was not limited to “knowledge to action” itself but included other derivations and conceptualizations (i.e., “dissemination”; “knowledge translation”; “knowledge transfer and exchange”; “knowledge utilization”). Team members and a University librarian were consulted and models or frameworks were decided to be identified in three ways:

1. a title and abstract keyword search in four prominent health science or general databases (Ovid MEDLINE(R), PsycINFO, AMED Allied and Complementary Medicine and EBSCO Host CINAHL), and on Google Scholar;
2. a review of the reference lists and cited articles of identified papers; and
3. discussions with experts in the field

**Fig. 1:**
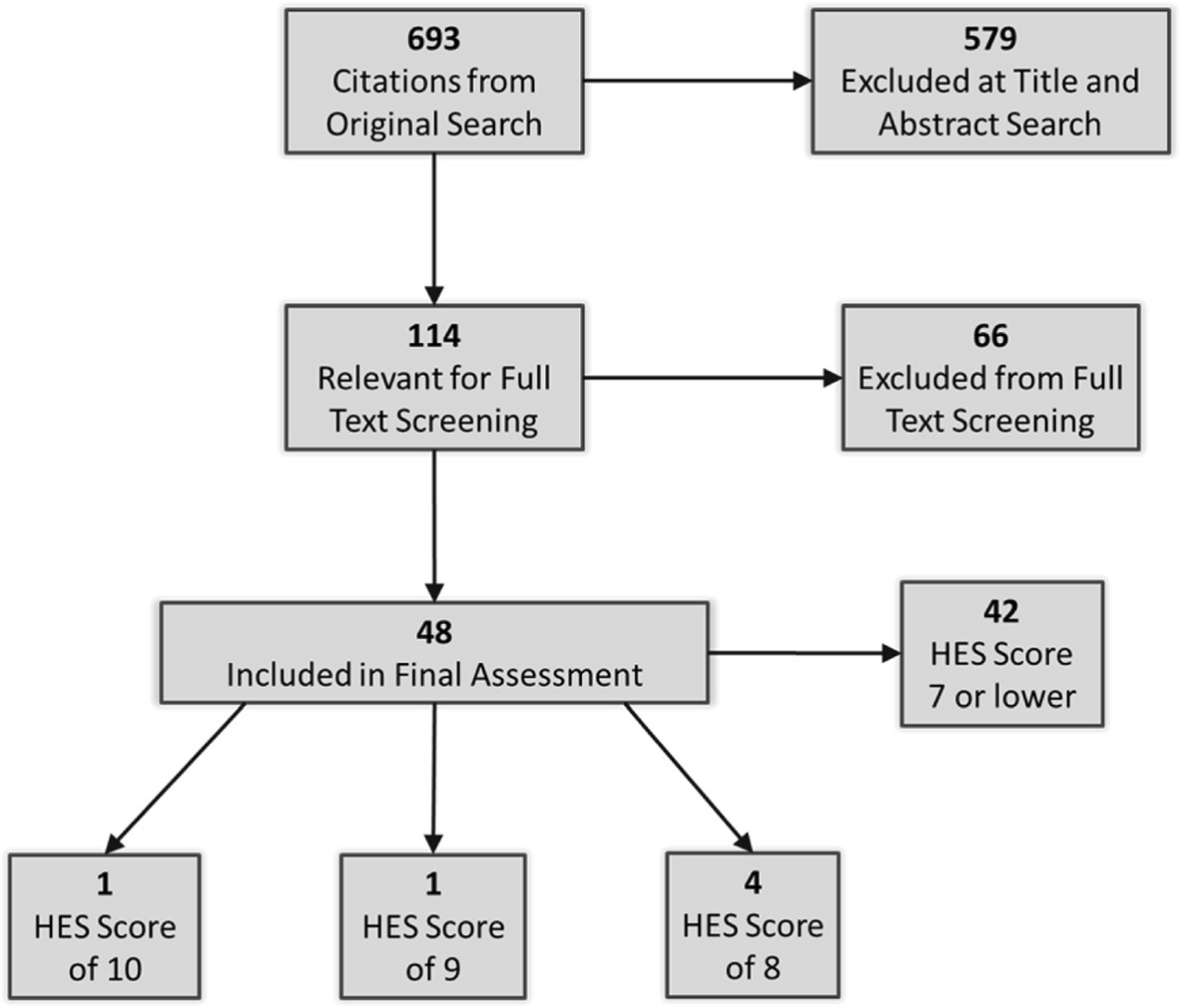
Search flow diagram

Modifications of the following search string were used for the database searches: (“dissemination” or “knowledge to action” or “knowledge translation” or “knowledge transfer”).mp. and (“model” or “framework”).m_titl. The database searches were limited by year (1997-present) and language (English). Articles were included if they contained a description, discussion, or critique of a specific model or framework for some derivation of “knowledge to action”. Results of this search strategy are outlined in Table 1. Titles and abstracts were reviewed for all identified papers by CMD and AP, those deemed potentials for inclusion were reviewed in full text by CMD and AP. The search flow diagram is outlined in Fig. 1.

**Table 1:** Search location and results

Search Location	Search Results
Ovid MEDLINE(R) 1944 to August Week 2 2012	464 relevant documents
PsychINFO 1967 to August Week 2 2012	89 additional documents
AMED (Allied and Complementary Medicine) 1985 to August 2012	3 additional documents
EBSCO Host CINAHL August 2012	89 additional documents
Google Scholars (first 6 results screens)	26 additional documents (no year limitation)
Review of reference list of identified documents	18 additional documents (no year limitation)
Expert consultation	4 additional documents (no year limitation)

The search strategy identified a total of 693 relevant documents. Upon further review, 114 documents were identified as including an introduction, discussion, or critique of a model or framework for some derivation of “knowledge to action”. A table of all identified models or frameworks was then constructed. When the list was compiled, each was critiqued by CMD, using the description of the model or framework that was included in the identified document for six characteristics related to health equity. These criteria speak to factors associated with health equity challenges and are informed by the World Health Organization (WHO) Commission on the Social Determinants of Health [1], as well as previous work by the National Collaborating Centre for Determinants of Health [2], and key stakeholders (identified by name and organization in the acknowledgement section). The equity support characteristics included:

1. a specific focus, mention or consideration of equity, equality, justice, disadvantaged or vulnerable groups;
2. an inclusive conceptualization of knowledge (beyond scientific research) that ensures that different types of knowledge and/or ways of knowing might be considered in the evidence-base;
3. community members are represented and/or community participation is an explicit part of the model or framework;
4. interactions are supported across disciplines or sectors;
5. there is specific referral to the social, physical, political, and/or economic context of knowledge generation and use; and/or
6. there is an applied, proactive or problem-solving focus.

The models were assessed using a three-point scale 0 = no obvious mention or inclusion in the description available, 1 = some or partial mention or inclusion, and 2 = this characteristic was clearly reflected. A total score was then calculated. This total score is referred to as the “health equity support” (HES) score. The results of this assessment can be found in Table 1.

## Findings

### Overview

In total, 48 unique models or frameworks for knowledge to action were found (Table 2). The majority of examples were from the last 15 years (as the inclusion criteria prioritized articles from 1997 to present), however, the Internet search, scan of reference lists and consultations with experts were not date limited, and as such, some models do fall outside this time period. An additional Excel file provides a list of these models with a brief description for each [see Additional file 1: Table S1].

**Table 2:** Health equity analysis of knowledge to action models (rated on a scale where 0 = none, 1 = some/partial, and 2 = clearly reflected)

Model [Reference]	Explicit focus on equity or related value	Inclusive conceptualization of knowledge	Stakeholder engagement	Explicit focus on interactions across jurisdictions or sectors	Context emphasized	Applied, pro-active, problem-solving	Total HES Score
Knowledge Brokering Frameworks [21]	2	2	2	1	2	1	10
A Model for Knowledge Translation and Exchange with Northern Aboriginal Communities [25]	2	2	2	0	2	1	9
A Framework for Research Transfer [22]	0	1	2	1	2	2	8
Joint Venture Model of Knowledge Utilization [23]	0	2	2	1	2	1	8
Translational Research Framework to Address Health Disparities [24]	2	2	0	0	2	2	8
Ecohealth Model Applied to Translate Knowledge [26]	2	1	1	2	2	0	8
Locally Based Research Transfer Model [35]	0	0	2	1	2	2	7
User-Context Framework for Knowledge Translation [36]	0	1	2	0	2	2	7
Promoting Action on Research Implementation in Health Services (PARIHS) Framework [37]	0	2	1	0	2	2	7
Equity-Oriented Knowledge Translation Framework [38]	2	1	0	0	2	2	7
The Knowledge Value Chain [39]	0	2	1	1	1	2	7
Model for Large-Scale Knowledge Translation [40]	1	1	0	2	2	1	7
Ottawa Model of Research Use [41]	1	2	0	0	2	1	6
CHSRF Model of Knowledge Transfer and Exchange [42]	0	0	2	0	2	2	6
Replicating Effective Programs Framework [43]	0	1	2	0	1	2	6
The Sticky Knowledge Framework [44]	0	1	1	0	2	2	6
Tehran University of Medical Sciences (TUMS) Knowledge Translation Model [45]	0	1	1	0	2	2	6
Collaborative Model for Knowledge Translation between Research and Practice Settings [46]	0	1	2	0	1	2	6
Knowledge Translation as part of the Research Cycle Model [19]	1	0	2	0	2	1	6
Conceptual Model for Considering the Determinants of Diffusion, Dissemination, and Implementation [47]	0	0	2	0	2	2	6
Model of Strategic Change [48]	0	2	1	0	2	1	6
The Trinity Evidence-Based Practice Model [49]	0	2	0	0	2	2	6
Advancing Research and Clinical Practice through Close Collaboration (ARCC) Model of Evidence-Based Practice in Nursing and Healthcare [50]	0	0	2	0	2	2	6
Knowledge to Action Process Model [9]	0	2	0	0	2	2	6
Practical, Robust Implementation and Sustainability Model (PRISM) [20]	0	1	1	0	2	2	6
Framework for Transferring Knowledge into Action [51]	0	2	0	0	2	2	6
Four Levels of Knowledge Utilization [52]	0	1	0	0	2	2	5
ACE Star Model of Knowledge Transformation [53]	0	2	0	0	2	1	5
Pathman-PRECEDE Model for Knowledge Translation [54]	0	1	1	0	1	2	5
Framework for Translating Evidence into Action [55]	0	1	0	0	2	2	5
Diffusion of Innovations Model [56]	0	1	1	0	1	1	4
Two-Communities or Two-Cultures Model [57]	0	0	1	0	1	2	4
Framework for Changing Implementation Behaviour [58]	0	0	0	1	2	1	4
Technology Transfer Model [59]	1	1	0	0	1	1	4
Model of Research Utilization [60]	0	0	1	0	2	1	4
Five-Point Knowledge Translation Framework [61]	0	1	2	0	0	1	4
Reach, Efficacy or Effectiveness, Adoption, Implementation, Maintenance (RE-AIM) [62]	0	1	1	0	1	1	4
Outcomes-Focused Knowledge Translation Intervention Framework [63]	0	0	2	0	0	2	4
Stages of Research Utilization Model [64]	0	0	1	0	1	2	4
Interactive Systems Framework for Dissemination and Implementation [65]	0	2	2	0	1	1	4
Iowa Model of Evidence-Based Practice [66]	0	0	2	0	0	2	4
Six Knowledge Utilization Models [67]	0	1	1	0	1	0	3
Measuring Knowledge Utilization Model [68]	0	2	0	0	1	0	3
Research Utilization Model [69]	0	0	0	0	1	2	3
Framework for Research Dissemination and Utilization [70]	0	0	1	0	1	1	3
Translational Framework for Public Health Research [71]	0	0	0	0	0	2	2
A Model for Evidence- Based Practice Implementation [72]	0	0	0	0	2	0	2
Knowledge Translation within a Communication System Paradigm [73]	0	0	0	0	0	1	1

0 = no obvious mention or inclusion in the description available, 1 = some or partial mention or inclusion, 2 = this characteristic was clearly reflected

Overall, there is a great variety among knowledge to action models or frameworks. These vary in the way they define and conceptualize knowledge (e.g. research evidence, innovations, ideas) and knowledge to action (e.g. translation, transfer, evidence-based practice, exchange, implementation etc.). They also differ in their point of focus. Some conceptualize the use of knowledge for tactical or political purposes (models 22, 23 and 72 for example); others focus on the interactions, barriers and facilitators that are involved when knowledge is to be used to inform decisions (models 25, 42 and 59 for instance). Some are problem solving and applied in nature (such as models 40, 58 and 63), while others are more theoretical and philosophical in their approach (such as models 47, 51, 56 and 57). It does appear that there has been some evolution in the field over the past 15 years. For example, our analysis identified at least four examples of models or frameworks that have emerged or have built upon others over time (e.g. Canadian Health Services Research Foundation (CHSRF) – now the Canadian Foundation for Healthcare Improvement - Model of Knowledge Transfer and Exchange [18]; Canadian Institutes for Health Research (CIHR) Knowledge Translation in the Research Cycle Model [19]; Equity-Oriented Framework [8]; and Practical, Robust Implementation and Sustainability Model (PRISM) [20]. There also appears to be more consistent focus on the context of knowledge to action as time has progressed.

### Relevance of models to health equity

Table 1 lists the 48 models or frameworks that were identified and indicates their status with respect to six characteristics important in supporting health equity. Models could score a 0–2 value for each of the six equity variables. A total score (a maximum of 12) was then calculated and this was termed the “health equity support” or HES score. The HES score is indicated for all models and the top six HES scored models are highlighted. This approach was not meant to be definitive and exclusionary, but instead is used as a systematic way to help identify models or frameworks, among a large number, that may have particular relevance for health equity while at the same time reporting some information for each model or framework found.

The models with the highest HES scores are the Knowledge Brokering Frameworks [21]; the Framework for Research Transfer [22]; the Joint Venture Model of Knowledge Utilization [23]; the Translational Research Framework to Address Health Disparities [24]; the Model of Knowledge Translation and Exchange with Northern Aboriginal Communities [25]; and the Ecohealth Model applied to knowledge translation [26]. These six models are discussed in detail below.

The Knowledge Brokering Frameworks outlined by Oldham and McLean [21] scored highest on the knowledge to action - health equity assessment. These are a series of three frameworks for knowledge brokering: a knowledge framework, a transactional framework, and a social change framework. The combination of these three frameworks had important implications for its high ranking in the health equity assessment. It explicitly supports an inclusive conceptualization of knowledge and although there is some emphasis on research evidence, it is not limiting. It prioritizes the engagement of a variety of stakeholders, and it has a strong emphasis on contextual factors. In addition, it discusses how the use of a social change framework in knowledge brokering could help address power differentials and encourage work that supports human rights.

The next highest scoring model in the health equity assessment was the model of Knowledge Translation and Exchange with Northern Aboriginal Communities [25]. This model focuses on knowledge translation specifically for northern Indigenous peoples. Using this model would include: establishing partnerships and trust with and among community members; undertaking capacity development activities; and engaging community field workers in all stages of research planning, data collection, analysis, interpretation, and dissemination. Researchers are called to have regular workshops for all members of the research team and make a commitment to return research results to the participants and communities first for verification and validation. There is also a commitment to make research and policy products relevant so that government decision makers might use them to inform policy and practice. The authors propose a true gold standard for integrated research and knowledge translation with vulnerable groups and include a specific sensitivity to the added ethical, cultural and spiritual dimensions of knowledge translation with Indigenous peoples. This model scores high on the health equity assessment because it has an explicit focus on equity and justice; it reflects an inclusive conceptualization of knowledge; it promotes meaningful and prolonged community engagement; and it is sensitive to contextual factors. The model scores lower on the problem-solving variable; although it is implicitly an applied approach, the authors do not explicitly describe whether research is chosen (or should be chosen) based on a specific issue or problem, nor do they describe how that priority setting might be approached. The model also does not emphasize work across jurisdictions or sectors, although it would be possible to see how this could be easily integrated.

There is a further group of four models that scored “8” in the health equity assessment. The Translational Research Framework to Address Health Disparities proposed by Fleming et al. [24] is a framework that is specifically focused on addressing health disparities by better aligning and translating research. This framework is made up of two interlinked conceptual models. The first model illustrates how to advance health disparities research through identifying disparities, examining their causes, developing and implementing interventions, and monitoring differential outcomes. The second model outlines knowledge to action and the different components of this in all realms of health research (e.g. the translation of knowledge from “bench” to “bedside” or from “bedside” to “community and public health practice”). The authors emphasize the need to connect biomedical to public health and clinical research, and to use research for real-world applications and community health intervention. The strengths of this model are that it focuses specifically on issues of health disparities, and there is a logical consideration of addressing problems associated with these disparities. There is also significant emphasis placed on contextual factors and the authors support an inclusive idea of knowledge.

Two other models: A Framework for Research Transfer [3] and The Joint Venture Model of Research Utilization [23] also scored “8” in the health equity review, and for similar reasons. These models touch on most aspects of the six features examined and had a strong emphasis on contextual features. Edgar and colleagues focus upon the interactions that happen in particular contexts, for example, individuals as they engage in organizations that in turn exist in social environments. Leadership, emotional intelligence and work, and socio-political environments are all featured components as well. In their framework, Nieva and colleagues identify that end users need a “change leader”, and that intervention tools need to be adapted to local needs and to particular organizational contexts. Developments and adaptations of knowledge to action strategies for particular contexts of health equity could be supported by components of these models. In addition, both of these models have some reference to work across disciplines or sectors.

The final model to receive an “8” in the health equity assessment was the Ecohealth Model as applied to knowledge translation [26]; this is a combination of a health model and a knowledge to action model. The Ecohealth Model (described by Hancock [27] as well as others) links the fields of health and ecology and focuses on the health of humans, the health of other species, and the natural environment. Humans and human health are components of ecosystems. Arrendondo and Orozco [26] take this conceptualization and overlap it with a model of knowledge to action that includes the participation of researchers and other specialists in specific knowledge areas (and of different types of knowledge) with community members and other decision makers. The authors highlight that the pillars of transdisciplinarity, participation, and equity support an overlapped model of Ecohealth and knowledge to action.

## Discussion

### Review of findings

The purpose of this project was to identify existing knowledge to action models or frameworks and critically examine their utility for promoting or supporting health equity. Forty-eight knowledge to action models or frameworks were identified. All of the models were then assessed across six characteristics relevant for supporting health equity. While no models scored full marks, the highest scoring models were found to have features relevant to advancing health equity.

In the assessment, we propose six characteristics that could be important markers: 1) an explicit mention of equity, justice or similar concept; 2) the involvement of various stakeholders; 3) an explicit focus on engagement across multiple sectors or disciplines; 4) the use of an inclusive conceptualization of knowledge; 5) the recognition of the importance of contextual factors; and, 6) a proactive or problem-solving focus. Specific populations, topics and solutions are marginalized, ignored, or not acted upon when, for example, only certain knowledge is considered valuable, when we don’t have a specific focus on equity or justice, and when we don’t work across sectors or consider contextual determinants of health [1, 2].

### Assessment of what might be missing in the models or frameworks

Health inequities are often enduring and profound. Commonly, factors that lead to inequity are deeply embedded in systems, processes, and norms of societies and cultures [5]. In addressing the “causes of the causes” [28] of health inequities, multisectoral approaches, focused on recognizing and addressing inequities, have been heralded [1, 2]. Of the six health equity supportive characteristics looked for in the knowledge to action models an explicit mention of multisectoral approaches or actions in knowledge translation was largely absent with only one model within the top six, the Ecohealth Model Applied to Translate Knowledge [26], strongly demonstrating integration of this component.

In order to inform decisions and change situations of inequity, adopting, collecting, synthesizing or valuing various new pieces of knowledge is often required. This can require difficult shifts from norms of practice, current and ingrained behavior, or systems of engagement, especially if considering work that might span disciplines or sectors. The creation of supportive structures in this process is ideal [3]. Knowledge brokering involves guided actions that can link producers of knowledge, including knowledge about inequities, with possible knowledge users [29]. This is sometimes conceptualized by focusing on guided interactions between researchers and decision makers [18] where these two groups are largely situated in different realms or communities. Knowledge brokers, whether whole organizations or specific individuals or groups, help to facilitate interactions; their goal is to support understanding and relationship building among diverse stakeholders. When a more full understanding of the various goals and professional cultures is established, new partnerships can be forged. This provides an opportunity for decisions to be informed by research knowledge [18, 30]. The Knowledge Brokering Frameworks outlined by Oldham and McLean [21] scored highest for supporting health equity among the 48 models identified. Included in this model are a knowledge framework, a transactional framework, and a social change framework. These frameworks explicitly support an inclusive conceptualization of knowledge; recognize the importance of contextual determinants of knowledge to action as well as the engagement of a variety of stakeholders. They have been designed to consider the social contexts and disrupt the power differentials that are at the heart of health inequities. Knowledge brokering has been an approach supported by Canadian organizations previously, including the Canadian Health Services and Research Foundation (currently the Canadian Foundation for Healthcare Improvement) and the Canadian Coalition for Global Health Research. It may be time to revisit this concept and these approaches when considering further actions for knowledge translation, health equity and the social determinants on health. It is not clear exactly how knowledge brokering could best be approached to ensure more effective action to address health inequity, however, this area represents an avenue for further discussion and scholarship as well.

### Recognized strengths in current models or frameworks

Links between vulnerability of specific populations and factors of social and physical environments are clear [31, 32]. There are many examples of models or frameworks where knowledge to action is conceptualized as holistic and interconnected and where features of context and environment are highlighted as important health and health equity determinants. Environmental features differentially impact sub-populations, and thus privilege some concerns or issues over others [33]. For this reason, models such as the Ecohealth Model [26] or the model of Knowledge Translation and Exchange with Northern Aboriginal Communities [25] have appeal. They have utility when considering the dynamic social, cultural, and historic features [33] surrounding knowledge to action work. The Jardine and Furgal [25] model, emanating from a community-based partnership for indigenous health research, has a strong emphasis on and recognition of the cultural, social, spiritual and geographic contexts; various types of knowledge and ways of knowing; and the essential nature of stakeholder engagement and leadership. Similarly, it is helpful to look towards research ethics models that have been developed for work with indigenous people [34] to further consider how participatory, culturally sensitive, integrative, or community-based approaches may be useful to inform knowledge to action and health equity pursuits. These models have not yet been used widely outside indigenous communities, and there remains distinct potential for their uptake in different arenas of action to advance health equity.

## Limitations

Our intention was to identify a group of models, especially those referred to over the past 15 years, in order to determine which ones may have utility in supporting health equity efforts. We understand that every existing model was not identified and that our assessment process was not formally validated. We searched only four prominent databases and we did not contact study authors for additional unpublished information about the different models or frameworks. We did not search “evidence-based” as a unique keyword (as in evidence-based medicine; evidence-based practice), however, we did include these types of models in our list if they were found in the documents amassed in the search strategy outlined in Fig. 1.

The characteristics making up the “heath equity assessment” score used to assess the models were generated from the literature and from discussions with key stakeholders (listed in the acknowledgements for this paper). Each of the characteristics were given the same weight. The assessment relied on the model descriptions which were often only briefly included in the literature. This may not accurately capture all aspects and nuance of health equity support, and does not always take into account how effectively a model can be applied in practice. The assessment was completed by just one person (primary author). We recognize that there may have been some variation in assessments if done by multiple independent reviewers, especially in interpretations of “partial” and “clearly” reflected.

## Conclusion

Forty-eight models of knowledge to action were identified and assessed based on six characteristics of health equity; the highest score being a possible 12. There was no single “perfect” model. Six models, all scoring between 8 and 10 of a maximum 12 points, exist as promising examples of knowledge to action models that may have utility for supporting health equity. Each could be strengthened in some way to make them more useful in supporting health equity by considering the six characteristics used in this review. Of particular interest is knowledge brokering as well as the use of holistic and cross-sector models of knowledge to action that consider environmental and contextual determinants. These are specific future avenues identified in this project. As there was no single ideal model found, discussion could also centre on what an ideal health equity, knowledge to action model might look like and if thought beneficial, how this could be developed, tested and used effectively. This conversation has been recently taken up by others [35] and this discussion could be further informed by those with knowledge and experience in knowledge brokering, as well as with Ecohealth approaches, and participatory and integrative research, and knowledge translation with Indigenous people.

## Implications for public health

- Existing knowledge translation models can help guide the application of knowledge to inform public health action to improve health equity. The six models analysed in detail exist as promising examples of knowledge to action models that have utility for supporting action on the social determinants of health and improving health equity.
- The most relevant models are those which embody principles and values reflective of equity and social justice.
- These models explicitly identify equity as a goal; value the involvement of various stakeholders; prioritize multisectoral engagement; use an inclusive conceptualization of knowledge; recognize the importance of contextual factors; and have a proactive or problem-solving approach.
- There is room to develop and test more robust equity supporting models. This conversation will require attention to the criteria proposed in this paper.

## Additional file

Additional file 1: Table S1. Description of Each Identified Knowledge to Action Models. Description of data: A tabulated summary of the 48 KTA models identified and evaluated including a brief description of each model.

## Abbreviations

ACE: Academic Center for Evidence-Based Practice
ARCC: Advancing Research and Clinical Practice through Close Collaboration
CHSRF: Canadian Health Services Research Foundation (currently the Canadian Foundation for Healthcare Improvement)
CIHR: Canadian Institutes for Health Research
EBP: Evidence-based practice
HES: Health equity support
KTA: Knowledge to action model
OMRU: Ottawa Model of Research Use
PARIHS: Promoting Action on Research Implementation in Health Services
PRECEDE: Predisposing, Reinforcing, and Enabling Constructs in Educational Diagnosis and Evaluation
PRISM: Practical, Robust Implementation and Sustainability Model
RE-AIM: Reach, Efficacy or Effectiveness, Adoption, Implementation, Maintenance
WHO: World Health Organization
